# Helminth infection is associated with dampened cytokine responses to viral and bacterial stimulations in Tsimane forager-horticulturalists

**DOI:** 10.1093/emph/eoab035

**Published:** 2021-10-26

**Authors:** India A Schneider-Crease, Aaron D Blackwell, Thomas S Kraft, Melissa Emery Thompson, Ivan Maldonado Suarez, Daniel K Cummings, Jonathan Stieglitz, Noah Snyder-Mackler, Michael Gurven, Hillard Kaplan, Benjamin C Trumble

**Affiliations:** 1 Center for Evolution and Medicine, Arizona State University, Tempe, AZ, USA; 2 Department of Anthropology, Washington State University, Pullman, WA, USA; 3 Department of Anthropology, University of California Santa Barbara, Santa Barbara, CA, USA; 4 Department of Anthropology, University of New Mexico, Albuquerque, NM, USA; 5 Tsimane Health and Life History Project, San Borja, Bolivia; 6 Economic Science Institute, Chapman University, Orange, CA, USA; 7 Institute for Advanced Study in Toulouse, Toulouse, France; 8 School of Life Sciences, Arizona State University, Tempe, AZ, USA; 9 School of Human Evolution and Social Change, Arizona State University, Tempe, AZ, USA

**Keywords:** soil-transmitted helminths, viruses, bacteria, cytokine storms, eosinophilia, hypereosinophilia, immunomodulation, hygiene hypothesis, old friends hypothesis

## Abstract

**Background:**

Soil-transmitted helminths (STHs) and humans share long co-evolutionary histories over which STHs have evolved strategies to permit their persistence by downregulating host immunity. Understanding the interactions between STHs and other pathogens can inform our understanding of human evolution and contemporary disease patterns.

**Methodology:**

We worked with Tsimane forager-horticulturalists in the Bolivian Amazon, where STHs are prevalent. We tested whether STHs and eosinophil levels—likely indicative of infection in this population—are associated with dampened immune responses to *in vitro* stimulation with H1N1 and lipopolysaccharide (LPS) antigens. Whole blood samples (*n* = 179) were treated with H1N1 vaccine and LPS and assayed for 13 cytokines (INF-γ, IL-1β, IL-2, IL-4, IL-5, IL-6, IL-7, IL-8, IL-10, IL-12p70, IL-13, GM-CSF and TNF-ɑ). We evaluated how STHs and eosinophil levels affected cytokine responses and T helper (Th) 1 and Th2-cytokine suite responses to stimulation.

**Results:**

Infection with *Ascaris lumbricoides* was significantly (*P* ≤ 0.05) associated with lower response of some cytokines to H1N1 and LPS in women. Eosinophils were significantly negatively associated with some cytokine responses to H1N1 and LPS, with the strongest effects in women, and associated with a reduced Th1- and Th2-cytokine response to H1N1 and LPS in women and men.

**Conclusions and implications:**

Consistent with the ‘old friends’ and hygiene hypotheses, we find that STHs were associated with dampened cytokine responses to certain viral and bacterial antigens. This suggests that STH infections may play an essential role in immune response regulation and that the lack of STH immune priming in industrialized populations may increase the risk of over-reactive immunity.

**Lay Summary:** Indicators of helminth infection were associated with dampened cytokine immune responses to *in vitro* stimulation with viral and bacterial antigens in Tsimane forager-horticulturalists in the Bolivian Amazon, consistent with the ‘old friends’ and hygiene hypotheses.

## INTRODUCTION

Infection with soil-transmitted helminths (STHs) can modulate the host immune response and ultimately shape morbidity and mortality associated with viral and bacterial infections [1, 2]. The vast majority of human history occurred in environments characterized by high STH prevalence, which has fundamentally shaped the evolution of human immunity and created a potentially essential role for STHs in regulating immunity in the face of co-infections [3–5]. Indeed, the mismatch between high STH rates over human evolutionary history and their near absence in many contemporary industrialized communities has been linked to immune dysregulation resulting in over-reactive immune function and allergy [5–7], while the presence of STHs in more rural communities has been hypothesized to play a protective role against runaway inflammatory immune responses [4, 5, 8]. This is suggested to be a product of a coevolutionary trajectory that has led to relatively low helminth virulence via parasite-induced manipulation of the host immune system [1, 5]. These relationships are often conceptualized as the ‘hygiene hypothesis’ and the ‘old friends’ hypothesis.

The hygiene hypothesis argues that there is a mismatch between current sanitary urban environments and the conditions in which the majority of human evolution occurred, such that the immune systems of many contemporary urban populations that evolved to cope with high levels of pathogen exposure instead experience low pathogen exposure throughout their lives. This lack of immune stimulation and priming results in dysregulated adult immune function and gives rise to increased rates of allergy and autoimmune infection [5]. The ‘old friends’ hypothesis builds on the hygiene hypothesis, focusing specifically on the co-evolutionary role of macroparasites in priming T-helper type 2 (Th2) immune activation [1]. STHs can catalyze trade-offs between Th1 and Th2 responses that can modulate immunological responses to other pathogens. The ‘old friends’ hypothesis suggests that the lack of co-evolved parasites and pathogens in contemporary urban environments releases the Th1 response from Th2 modulation or regulation, and that higher rates of autoimmune disorders and allergies in industrialized populations can be attributed to a lack of helminth-induced moderation of reaction to self-antigens and allergens [1, 5, 7]. Lack of STH immune priming may also increase the risk of overreaction to mild or moderate viral and bacterial infections, which has been hypothesized to increase morbidity and mortality associated with conditions such as COVID-19 [4, 8]. Together, both hypotheses center on the idea that changes in pathogen exposure affect immune function in ways that shape disease susceptibility. However, because most medical research takes place in industrialized urban environments, our understanding of potentially deleterious and protective interactions between STHs and immune function is limited.

STHs elicit Th2-polarized immune responses in the simplified Th1/Th2 paradigm [9, 10]. Within this paradigm, naive CD4+ cells are expected to differentiate into cells with distinct functions based on antigen presentation [11]. Intracellular parasites (e.g. protozoa, viruses) typically trigger Th1 responses that activate a broadly proinflammatory response (e.g. interferon gamma (IFN-γ), interleukin-2 (IL-2)). Helminths trigger Th2 responses that activate cells to release anti-inflammatory and regulatory cytokines, which mediate the activation of effector mechanisms that include the antibody-based immune response and regulatory T cells [9, 12, 13], inhibit the proinflammatory Th1 response, and modulate antigen reactivity [14–16]. Helminths may thus be powerful modulators of the immune response to subsequent infections.

Among the most devastating outcomes of certain viral and bacterial infections is the induction of a hyperactive cytokine response (‘cytokine storm’) that can cause tissue and organ damage and is associated with many of the deaths in viral outbreaks, including the current COVID-19 pandemic [8, 17]. Cytokine storms are characterized by an unfettered increase in proinflammatory cytokines [18] and are associated with a greater incidence of severe illness and mortality [17, 19]. Because STHs can inhibit proinflammatory Th1 responses and promote regulatory T cells [16, 20], they may, paradoxically, provide protection against some of the worst outcomes of viral and bacterial infections.

Understanding how STHs affect downstream viral and bacterial infections is particularly crucial for non-industrialized or rural populations with high infection prevalence. Over 12% of the world’s population is infected with at least one STH [21] and the spread of highly contagious viral and bacterial infections continues to accelerate via globalization and international travel [22]. Thus, understanding the ways in which STHs affect the immunological response to and outcomes of viral and bacterial infections is particularly exigent.

This study focuses on STHs and immune response in Tsimane forager-horticulturalists of the Bolivian Amazon, who practice a largely non-industrial lifestyle centered around hunting, fishing, gathering and small-scale slash-and-burn farming, and have relatively little interaction with market economies [23–25]. The Tsimane have high STH rates (up to 76% [26, 27]) which is likely linked to the lack of sanitation infrastructure, inconsistent access to footwear, shared space with animals and open defecation practices [23, 28, 29]. We assess the hypothesis that STHs inhibit the cytokine response to viruses and bacteria by dampening the proinflammatory cytokine phenotype using whole blood stimulation with H1N1 vaccine and lipopolysaccharide (LPS) antigens. LPS is a component of gram-negative bacteria cell walls that induces an innate immune response characterized by inflammation in stimulations [30]. The H1N1 vaccine allows for viral stimulation without using live virus in the absence of a completely controlled laboratory setting. At the time of data collection, H1N1 was not known to have spread in the area and thus would represent a novel strain that the participants had not yet encountered.

We evaluate the responses of 13 cytokines and two functional cytokine suites (e.g. pro- and anti-inflammatory) to H1N1 vaccine and LPS as a function of two metrics related to parasitism: the presence of each of the five most common STHs in this population and eosinophil count, a measure of white blood cell activation that is part of the STH immune response. We evaluate these patterns separately in women and men, predicting that metrics of parasitism will be associated with lower proinflammatory immune responses and that women will exhibit stronger proinflammatory responses than men based on higher female immune responsiveness (e.g. increased immune cell proliferation and activation, upregulation of immune-specific genes) [7, 31, 32]. All predictions are summarized in Table 1.

**Table 1.** Predictions, expected cytokine effects, and results

Prediction	Predicted effects	Results (H1N1 vaccine)	Results (LPS)
STH presence/absence associated with lower proinflammatory immune response to H1N1 vaccine and LPS stimulation	↓IFN-γ, IL-2; ↓Th1-suite; women > men	Women:	Women:
		↓ IL-1β, IL-2, IL-6, IL-7, IL-10, IL-13, GM-CSF (*A. lumbricoides* only)	↓ IFN-γ and IL-7 (*A. lumbricoides* only)
			↓ Th1-suite
		Men: none	Men: ↓ IFN-γ (hookworm sp. only)
Eosinophil count associated with lower proinflammatory immune response to H1N1 vaccine and LPS stimulation	↓ IFN-γ, IL-2; ↓ Th1-suite; women > men	Women:	Women:
		↓ IFN-γ, IL-1β, IL-2, IL-4, IL-7, IL-8	↓ IFN-γ, IL-4, IL-6, IL-7, IL-8, GM-CSF
		↓ Th1-suite	↓ Th1-suite
		Men: ↓ IFN-γ, IL-2	Men: ↓ IFN-γ, IL-1β, IL-6, IL-7, IL-8
		↓ Th1-suite	↓ Th1-suite

## METHODS

### The Tsimane Health and Life History Project

The Tsimane Health and Life History Project (THLHP) has worked with a population of approximately 16 000 Tsimane across ∼90 villages since 2002, providing routine medical care and collecting epidemiological and biodemographic data [23]. Medical care is provided to all individuals and informed consent is obtained from individuals, villages and the Tsimane government (Gran Consejo Tsimane). All research is approved by the University of New Mexico and University of California Santa Barbara Institution Research Boards (IRB # 07-157, 15-133). None of the individuals who participated in this study had received anthelmintic treatment (i.e. mebendazole, albendazole) or antibiotics from our medical staff within at least 8.5 months (mean for the 71% of sampled individuals with a record of treatment = 1.4 years) of sample collection beginning in March 2011. To our knowledge, no Tsimane had ever been vaccinated against H1N1 or other viruses at the time of this study.

### Biomarker data collection

Biomarker data were collected from 179 Tsimane adults who participated in an antigen stimulation study. This sample included 82 women aged 29–71 (median age: 46.5) and 97 men aged 37–89 (median age: 49). Six women were pregnant at the time of the study (based on back-calculating from next birth). Participants attended the THLHP Clinic in San Borja, Bolivia for routine medical care and biodemographic data collection between March-November 2011. Fasting morning blood (5 ml) was collected into a heparinized vacutainer tube. A manual white blood cell count and five-part differential (including eosinophils) were conducted with a hemocytometer immediately following each blood draw, and fecal samples were collected for parasite identification using fecal smear microscopy or a density separation technique [28].

### Helminth infections

We characterized parasite communities by identifying parasite species with direct smear (30% of samples) microscopy [27] as well as Percoll^®^ separation (70% of samples) in fresh fecal samples [2]. We placed all hookworm eggs in a single category because the eggs of hookworm species can be difficult to differentiate morphologically. We combined the results of both methods for all analyses, and assessed co-occurrence of parasite species with a Pearson’s correlation matrix.

### Eosinophils

Microscopic techniques such as direct smears and density separations can produce false negatives based on non-random distribution of eggs in feces and life cycles [33–35]. In addition, 30% of our fecal samples were not processed for quantitative egg counts (those processed with direct smears rather than Percoll separation), precluding estimation of infection intensity across the dataset. We thus performed additional analyses, first on the relationship between parasite infections and eosinophils, which occupy a cardinal role in the helminth immune response [36] and are a common indicator of STHs in clinical settings [37, 38]. We modeled eosinophils (cells/µl) as a function of each parasite (presence/absence) for species with >10% prevalence in generalized linear models (GLMs) with age included as a continuous predictor in each model (women/men). We then used eosinophil count as a proxy of parasitism in downstream analyses.

Eosinophils can be associated with allergies or autoimmune disorders in industrialized populations; however, as in many non-industrialized populations [1], virtually no allergies or autoimmune disorders have been identified among the Tsimane [26]. This absence may arise from the regulatory immunophenotypes induced by helminths (indeed, antihelminthic treatment is associated with lower eosinophils in high STH prevalence areas) [39, 40], and suggests that—while we cannot rule out eosinophilic leukemia or other conditions—eosinophils are likely to indicate helminth infection in the Tsimane. We use eosinophil count here as a secondary metric to the presence/absence of each STH that may capture additional elements of infection, including infection intensity or periods of migratory larval stages characterized by pre-reproductive larvae that do not yet produce eggs.

### *In vitro* antigen stimulation

Aliquots of 100 µl heparinized whole blood were immediately added to separate round bottom microtiter wells in a sterile 96-well plate. One aliquot received 1 µg/ml H1N1 vaccine [2009 Monovalent Vaccine (Sanofi Pasteur, Inc. Swiftwater PA 18370)] diluted in RPMI-1640. Another aliquot received 100 µl of 20 µg/ml LPS (Sigma cat. L2630) diluted in RPMI-1640, rendering a final concentration of 10 mg/ml LPS. Control aliquots were run in RPMI alone. To prevent contamination, RPMI was supplemented with 100 IU/ml penicillin and 100 µg/ml streptomycin (Sigma cat. P0781), and any plates with visible growth or control values indicating contamination were eliminated. We enriched CO_2_ concentration by sealing plates in an airtight container with a burning candle to deplete O_2_ as a field-friendly alternative to a CO_2_ incubator [41]. The sealed and treated blood samples were incubated at 37°C for 72 h. Samples were centrifuged and supernatants were frozen in liquid nitrogen, transported on dry ice, and stored at −80°C for up to 2 years (see [42, 43]).

At the Hominoid Reproductive Ecology Laboratory at the University of New Mexico, 13 cytokines (INF-γ, IL-1β, IL-2, IL-4, IL-5, IL-6, IL-7, IL-8, IL-10, IL-12p70, IL-13, Granulocyte-macrophage colony-stimulating factor (GM-CSF), and Tumor necrosis factor-alpha (TNF-ɑ)) were measured with a Milliplex MAP High-Sensitivity Human Cytokine Panel (HSCYTMAG-60SK-13, Millipore Corp., Billerica, MA, USA) on a Luminex MagPix (Millipore Corp., Billerica, MA, USA). All quality control specimens were within normal limits.

### Statistical analysis

#### Effect on individual cytokine responses

We assessed the impact of STHs on the response of each cytokine to stimulation with H1N1 and LPS antigens. Assessing women and men separately, we used GLMs to model the log-transformed concentration of each cytokine in either H1N1 of LPS as a function of the presence or absence of each STH. We included body mass index (BMI) and age as covariates in all models, and included a pregnancy covariate for women (0/1) [44]. The small number of pregnant women (*n* = 6) precluded a trimester-level analysis [45]. We included only STHs with >10% prevalence across the sample set.

We assessed the impact of eosinophil count (cells/µl) on individual cytokine response to stimulation. We used GLMs to model the response of log-transformed cytokines in response to stimulation with H1N1 and LPS by eosinophil count. We included BMI, age, and pregnancy for women, and included total leukocyte count to account for variation in total white blood cells. We assessed collinearity between eosinophil and leukocyte counts by examining the variation inflation factor (VIF) for all models; all VIFs were acceptably low (<2.5). We found no significant interaction between age and eosinophils and thus omitted this interaction from our final models.

We calculated a meta effect score by combining the fixed effects from each model for women and men (‘meta’ package [46]). Although this approach assumes cytokine independence, it allows for a broad estimation of the cross-cytokine effect of eosinophils and functions as a summary statistic.

We did not consider changes from baseline cytokine levels (unpublished data) because cytokines have short half-lives, ranging from minutes to hours [47]; thus, many of the circulating cytokines would have degraded during incubation and would not be present at biological relevant levels at the time of assay.

#### Effect on Th1–Th2 response

Because cytokines are pleiotropic, we created functional Th1 and Th2 categories according to standard designations. Generally, Th1-type cytokine groups include proinflammatory cytokines such as IFN-γ and IL-2 [48]. Likewise, Th2-type cytokine groups generally include anti-inflammatory or regulatory cytokines, such as IL-4, IL-5 and IL-13 [48]. Our categories were based on the most conservative designations; our Th1-suite included IFN-γ and IL-2, while the Th2-suite included IL-4, IL-5 and IL-13 [48]. We ran parallel analyses with broader categories (Supplementary Table S10). This included a proinflammatory suite (IFNy, IL-2, IL-6, IL-8, IL-12p70, TNFa) and an anti-inflammatory suite (IL-4, IL-5, IL-10, IL-13).

We added a constant (+1) to and log-transformed each cytokine value. We calculated *z*-scores for each of the functional suites within each treatment with the sum of the individual cytokines. Finally, we averaged *z*-scores within each category to produce *z*-scored suite responses for each sample. Assessing women and men separately, we first modeled *z*-scores within each treatment by each STH (presence/absence), BMI, age and pregnancy (for women). We then performed similar analyses modeling the effect of eosinophil count on Th1/Th2-suite *z*-scores, including the same covariates as predictors and total leukocyte count. All analyses were done in *R*.

## RESULTS

### Descriptives

Helminth richness ranged from 0 to 4 species per person (median: 1, Fig. 1, *n* = 179), and 87% of samples had at least one helminth infection. We identified hookworm (*Necator americanus* or *Ancylostoma duodenale*) (75.4% prevalence), *Ascaris lumbricoides* (21.8%), *Strongyloides stercoralis* (13.4%), *Trichuris trichiura* (5.6%) and *Hymenopolis nana* (0.6%). The protozoan *Giardia lamblia* (13.4%) and the amoeba *Entamoeba histolytica* (7.3%) were also identified. Coinfections (37%) were most likely to occur with *A. lumbricoides* and *T. trichiura* (Pearson correlation = 0.23, *P* < 0.01, Supplementary Table S1) and least likely to occur between *A. lumbricoides* and hookworms (Pearson correlation = −0.27, *P* < 0.01, Supplementary Table S1). Only helminths with a prevalence of >10% were included for downstream analyses (*A. lumbricoides*, *S. stercoralis* and hookworm species; Supplementary Table S2).

**Figure 1.**
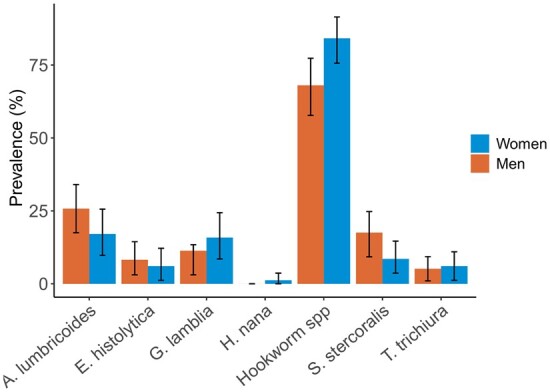
Prevalence of parasites identified microscopically in 179 fresh fecal samples

Eosinophil counts ranged from 492 to 7200 cells/µl, with a median of 1950 cells/µl and a mean of 2230 cells/µl. For comparison, the US reference range for eosinophils is <500 cells/µl; >500 cells/µl is categorized as eosinophilia [49]. Thus, by US standards all but two (98.6%) of the sampled Tsimane were eosinophilic.

### Parasites and eosinophils

The presence of *S. stercoralis* was the only parasite infection positively associated with increased eosinophils (*P* = 0.004) for men. No parasite infection was associated with changes in eosinophil count for women.

### Individual cytokine responses

#### H1N1 stimulation

**Figure 2.**
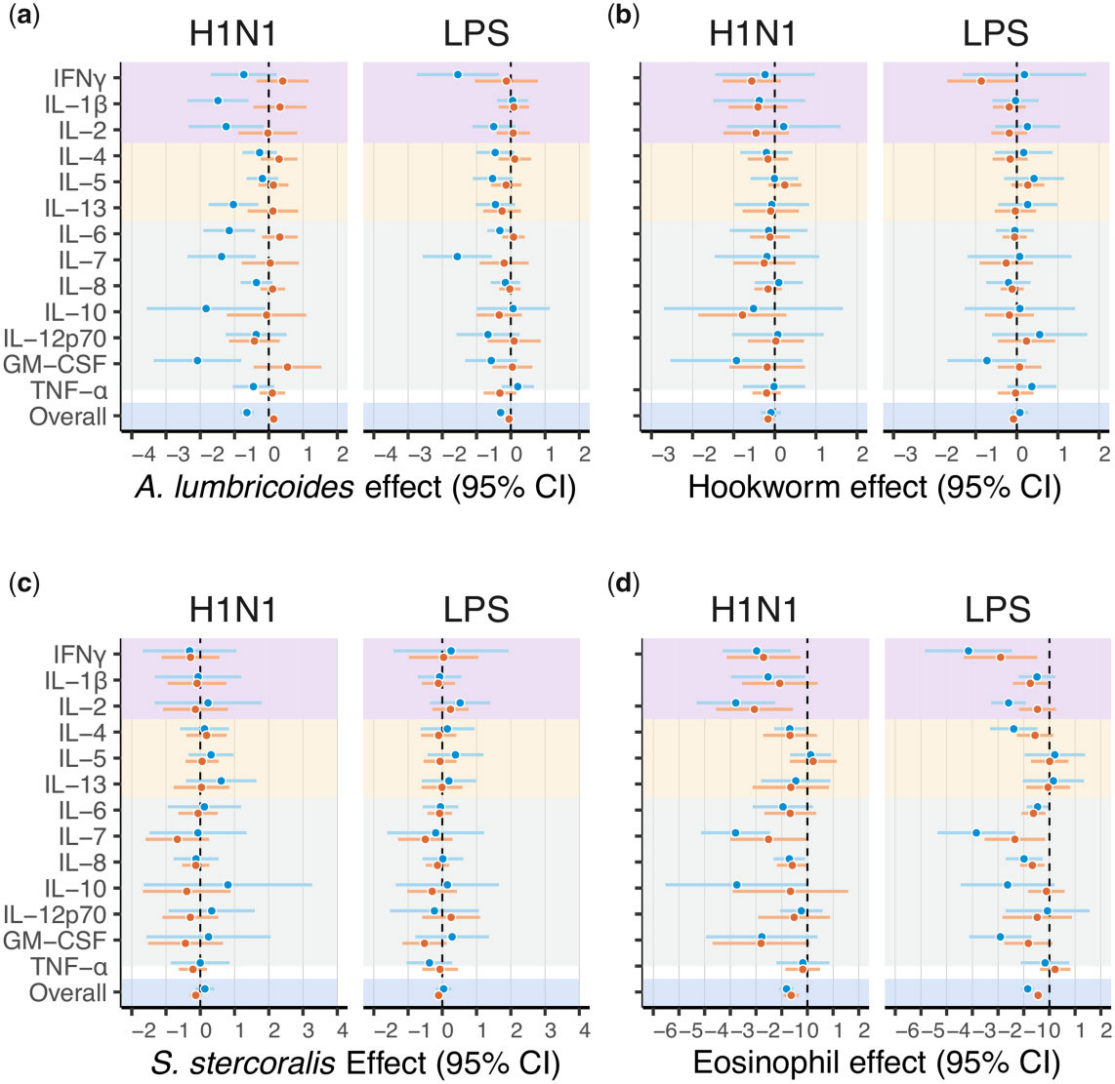
Individual cytokine responses to H1N1 and LPS stimulation as a function of (**a**) *A. lumbricoides*, (**b**) hookworms, (**c**) *S. stercoralis* and (**d**) eosinophil count. Coefficients and 95% confidence intervals are shown for each condition for women (blue) and men (orange). Cytokines are shaded by their Th1- (purple) or Th2-type (yellow) classification, and the overall effect size for each stimulation is highlighted in blue

For women, the presence of *A. lumbricoides* was significantly (*P* ≤ 0.05) negatively associated with the response of seven cytokines to H1N1 (IL-1β, IL-2, IL-6, IL-7, IL-10, IL-13 and GM-CSF; Supplementary Table S4). *Ascarislumbricoides* was the sole parasite to exhibit a significant relationship with cytokine responses for women, and exhibited negative betas associated with all 13 cytokines (Fig. 2 and Supplementary Table S4). The overall estimate of the impact of *A. lumbricoides* across cytokines for women in the fixed-effects model was −0.6 (*P* < 0.01; Fig. 2 and Supplementary Table S4). Eosinophils were significantly negatively associated with the response of six cytokines to H1N1 (IFN-γ, IL-1β, IL-2, IL-4, IL-7, IL-8), with eosinophils exhibiting negative betas for all of the 13 total cytokines but IL-5 (Fig. 2 and Supplementary Table S5). The overall estimate of eosinophil impact across cytokines for women in the fixed-effects model was −0.8 (*P* < 0.01; Fig. 2 and Supplementary Table S4). Age was positively associated with TNF-alpha and GM-CSF responses to H1N1 in parasite-specific models (Supplementary Table S5).

For men, individual parasite infections were not significantly associated with cytokine responses to H1N1 stimulation (Supplementary Table S6). Eosinophils were significantly negatively associated with the response of two cytokines to H1N1 (IFN-γ and IL-2), and exhibited negative betas for all cytokines but IL-5 (Fig. 2 and Supplementary Table S7). The overall estimate of eosinophil impact across cytokines in the fixed-effects model was −0.63 (*P* < 0.01; Fig. 2 and Supplementary Table S7). Age, BMI, pregnancy, and leukocyte count had varying effects on cytokine response to H1N1 in all models (Fig. 2 and Supplementary Table S7).

#### LPS stimulation

For women, the presence of *A. lumbricoides* was significantly negatively associated with the response of two cytokines to LPS stimulation (IFN-γ and IL-7; Supplementary Table S4). No other parasite infection demonstrated any relationship with cytokine expression. The overall estimate of the impact of *A. lumbricoides* on cytokine response in the fixed-effects model was −0.3 (*P* < 0.01; Supplementary Table S4). Eosinophils were significantly negatively associated with the response of seven cytokines to LPS (IFN-γ, IL-4, IL-6, IL-7, IL-8 and GM-CSF), and produced negative betas for all cytokines except IL-5 and IL-13 (Fig. 2 and Supplementary Table S5). The overall estimate of eosinophil impact across cytokines in the fixed-effects model was −0.9 (*P* < 0.01; Supplementary Table S5). Age, BMI, pregnancy, and leukocyte count had varying effects on cytokine responses to LPS. Age was positively associated with TNF-alpha and GM-CSF responses to LPS in parasite-specific models (Supplementary Table S5). Additional models excluding pregnant women were not substantially different from the primary models.

For men, the presence of hookworms was significantly negatively associated with the expression of IFN-γ to LPS stimulation (Supplementary Table S6). No other parasite infection exhibited any relationship with cytokine expression. Eosinophils were significantly negatively associated with the response of five cytokines to LPS (IFN-γ, IL-1β, IL-6, IL-7 and IL-8), and produced negative betas for all cytokines except IL-5 and TNF-ɑ (Fig. 2 and Supplementary Table S7). The overall estimate of eosinophil impact across cytokines in the fixed-effects model was −0.44 (*P* < 0.01) for men (Fig. 2), and age, BMI, and leukocyte count had varying effects on cytokine response to LPS. Specifically, age was associated with higher IL-7 response to LPS stimulation (Supplementary Table S7).

### Th1–Th2 responses

**Figure 3.**
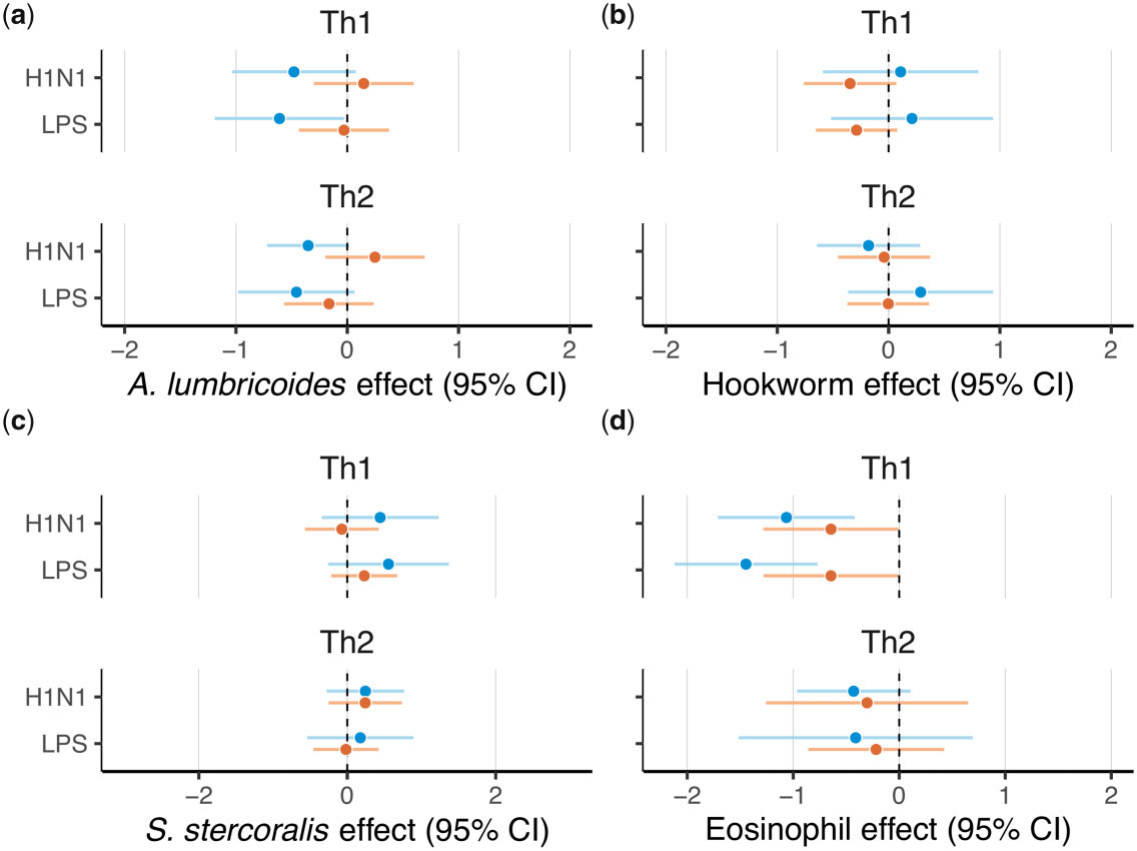
The effect of (**a**) *A. lumbricoides*, (**b**) Hookworm spp., (**c**) *S. stercoralis* and (**d**) eosinophils on Th1- and Th2-type responses to LPS and H1N1 stimulation. Coefficients and 95% confidence intervals (CIs) are shown for each condition for women (blue) and men (orange)

For women, *A. lumbricoides* was not associated with the Th1- or Th2-type cytokine suite to H1N1 stimulation, and was negatively associated with the response of the Th1- type cytokine suite (β = 0.6, *P* = 0.04) to LPS stimulation but not with the response of the Th2-type cytokine suite (Supplementary Table S8). Eosinophil counts were negatively associated with the response of Th1-type (β = −1.06, *P* < 0.01) but not Th2-type (β = −0.43, *P* = 0.13) cytokines to H1N1, with the same pattern observed in response to LPS stimulation (Th1-type: β = −1.45, *P* < 0.01, Th2-type: β = −0.41, *P* = 0.47, Fig. 3 and Table S9).

For men, no parasites were significantly associated with the response of either cytokine suite to stimulation with either H1N1 or LPS. Eosinophil counts were negatively associated with response of Th1-type (β = −1.13, *P* = 0.01) but not Th2-type cytokines (β = −0.3, *P* = 0.54; Fig. 3 and Supplementary Table S8) to H1N1 stimulation. Similarly, eosinophil counts were associated with the response of Th1-type (β = −0.64, *P* = 0.05) but not Th2-type cytokines (β = −0.22, *P* = 0.51; Fig. 3 and Supplementary Table S9) to LPS stimulation. The other predictors had varying effects on the expression of Th1- and Th2-type cytokines for women and men. Higher age was associated with higher Th1 responses to H1N1 for women (a similar effect was observed for LPS but it did not meet the significance threshold); no association was observed in men in either medium (Supplementary Table S9).

## DISCUSSION

As suggested by evolutionary hypotheses (e.g. ‘old friends’ and hygiene hypothesis), STH infections (specifically, *A. lumbricoides*) are associated with dampened proinflammatory responses to acute viral and bacterial stimulations among Tsimane women. Eosinophils, considered here as a secondary indicator of infection, were also significantly associated with lower cytokine responses to H1N1 and LPS in women and men. Together, these results suggest that helminth infections may attenuate the proinflammatory response to viruses and bacteria with the strongest effect in women.

### Helminths may dampen the acute immune response to infection

STH infection in the Tsimane was high, with at least one helminth found in 87% and two or more helminths found in 37% of individuals. In conjunction with the high levels of immunoglobulin E (IgE) observed in the Tsimane [26, 28], high eosinophil levels suggest that STH prevalence is likely even higher than suggested by microscopy. The most common infections were with the gastrointestinal nematodes *A. lumbricoides*, *S. stercoralis*, *T. trichiura* and hookworm spp, which have all co-evolved with humans to elicit anti-inflammatory and regulatory immune responses characterized primarily by Th2 cytokine cascades [50–52] with some evidence for a role of Th1 cytokine responses [51]. These primarily anti-inflammatory immune responses can inhibit the ability of the immune system to launch proinflammatory responses in the face of some viral and bacterial pathogens.

The proinflammatory immune responses elicited by H1N1 and similar viruses are characterized by elevations in proinflammatory cytokines [53]. While a certain degree of responsiveness is vital to combating infection, a proinflammatory ‘cytokine storm’ can culminate in tissue and organ damage; indeed, deaths attributed to viral infections and certain gram-negative bacteria (e.g. *E. coli* [19, 54]) are typically associated with cytokine storms [17, 18]. In Tsimane women, infection with *A. lumbricoides* was significantly associated with impaired responses of certain cytokines implicated in cytokine storms [53, 55]. This suggests that Tsimane women infected with *A. lumbricoides* may be less susceptible to the development of cytokine storms during viral or bacterial infections. In Tsimane men, infection with hookworms was only associated with a diminished response of IFN-γ in LPS. In the Th1/Th2-suite analyses, the only observed effect was the dampening of the Th1-suite response to LPS in women by *A. lumbricoides.* The sex-specific effect of *A. lumbricoides* occurs despite fairly equal infection prevalence among women and men (Table 1), and may be tied to parasite-specific sex differences in immunity [56, 57] and the profiles of chronic versus acute infections (Fig. 3).

As expected based on both the limitations of traditional microscopy and the binary nature of our parasite infection data, eosinophils were negatively associated with more cytokines than were individual parasite species. In women, higher eosinophil count was associated with decreased expression of both pro- and anti-inflammatory cytokines. In men, the effect was similarly spread across anti- and proinflammatory cytokines. In the functional Th1/Th2-suite analyses, eosinophils were associated with inhibited expression of the Th1-suite for both women and men in all media.

Associations between eosinophils and cytokine expression diverged from those between parasite presence and cytokine expression (i.e. certain cytokines were associated with eosinophils, but not with a specific parasite, and vice versa). This may be due to the limitations of our parasite detection techniques, or may be tied to factors that were not explicitly quantified here but may be reflected in our eosinophil measure. As infections with *A. lumbricoides*, *S. stercoralis,* and hookworms all involve an initial larval migration, our eosinophil analyses may capture infections at various points along the infection timeline, including prior to the reproductive phase characterized by high egg counts that would be captured with microscopy. Eosinophils may also protect against new infections, represent the tail-end of recently cleared infections, or reflect early life exposure to helminths that would prime individuals for anti-inflammatory responses throughout their lives. These components of parasitism may underlie the relationship observed between antihelminthic treatment and eosinophil count in certain populations with high STH infection rates [39, 40]. Indeed, Tsimane have significantly elevated IgE by the age of 5, and cross-sectional studies suggest that IgE remains high across the life course [2] consistent with lifetime STH exposure and infection. These results suggest that eosinophils may reflect a to-be-determined component of STH infection and recapitulate the immunosuppressive associations in the STH analyses.

We found the strongest immunosuppressive associations in women, pointing to sex differences in immune function deviating from the expected pattern of immune hyperactivity in women [7]. Women are generally less susceptible to pathogens [58, 59] and mammalian females tend to host parasite infections at lower rates and with lower loads than males [60] (but see [61]), which have been linked to the proinflammatory properties of estradiol [62] and the immunosuppressive components and energetic costs of testosterone [42, 63]. However, women have historically incurred higher morbidity and mortality associated with viral pandemics (reviewed in [62, 42, 63]). Many of the data that have pointed to higher morbidity and mortality in women during viral pandemics, however, are from industrialized populations, which are typically characterized by low STH rates [3], as well as high autoimmune risk and low fertility [7]. Our results suggest that concurrent STH infections may mitigate the risk of overreaction to viral and bacterial infection and may buffer women from the otherwise higher likelihood of development of cytokine storms through suppression of cytokine responses.

### Advantages and limitations

Studies that include data from non-industrialized populations are relatively rare and provide knowledge impossible to produce in a laboratory, but carry certain limitations (e.g. prior H1N1 exposure is unlikely but possible, low sample size of pregnant women precluded trimester-level analysis, limited data on early-life exposure, no causality assessed). Most studies on immune responses, allergies, and autoimmune disorders take place in populations that are wealthy and industrialized: in other words, under conditions that diverge sharply from those under which humans evolved [64]. Broadening studies of health and disease to populations that do not fit the wealthy and industrialized mold contributes to a more robust understanding of human variation and of effects on health and disease that originate in mismatch driven by rapidly changing human landscapes.

## CONCLUSIONS

Altogether, our results support the hypothesis that STHs inhibit the cytokine response to viruses and bacteria by dampening the proinflammatory cytokine phenotype (consistent with lower vaccine immunogenicity associated with helminth infections [65]) and suggest that helminths may buffer the stronger immune responses of Tsimane women against runaway proinflammatory responses [32, 60]. These results add to a growing level of support for the ‘old friends’ and hygiene hypotheses, suggesting that STH infections may play an essential role in regulating immune function, and provide further evidence that the lack of STH infections in industrialized populations may increase the risk of over-reactive immune responses in the absence of STH immune priming [3–5]. Although we did not directly test this relationship, our results suggest that the helminth-induced anti-inflammatory immunomodulatory network may attenuate some of the most severe symptoms of viral infections such as SARS-CoV-2 [4, 8]. Variation in helminth prevalence may thus play a role in the complex network of community-specific factors contributing to global COVID-19 mortality patterns.

## SUPPLEMENTARY DATA

Supplementary data is available at *EMPH* online.

## Supplementary Material

eoab035_Supplementary_Data
